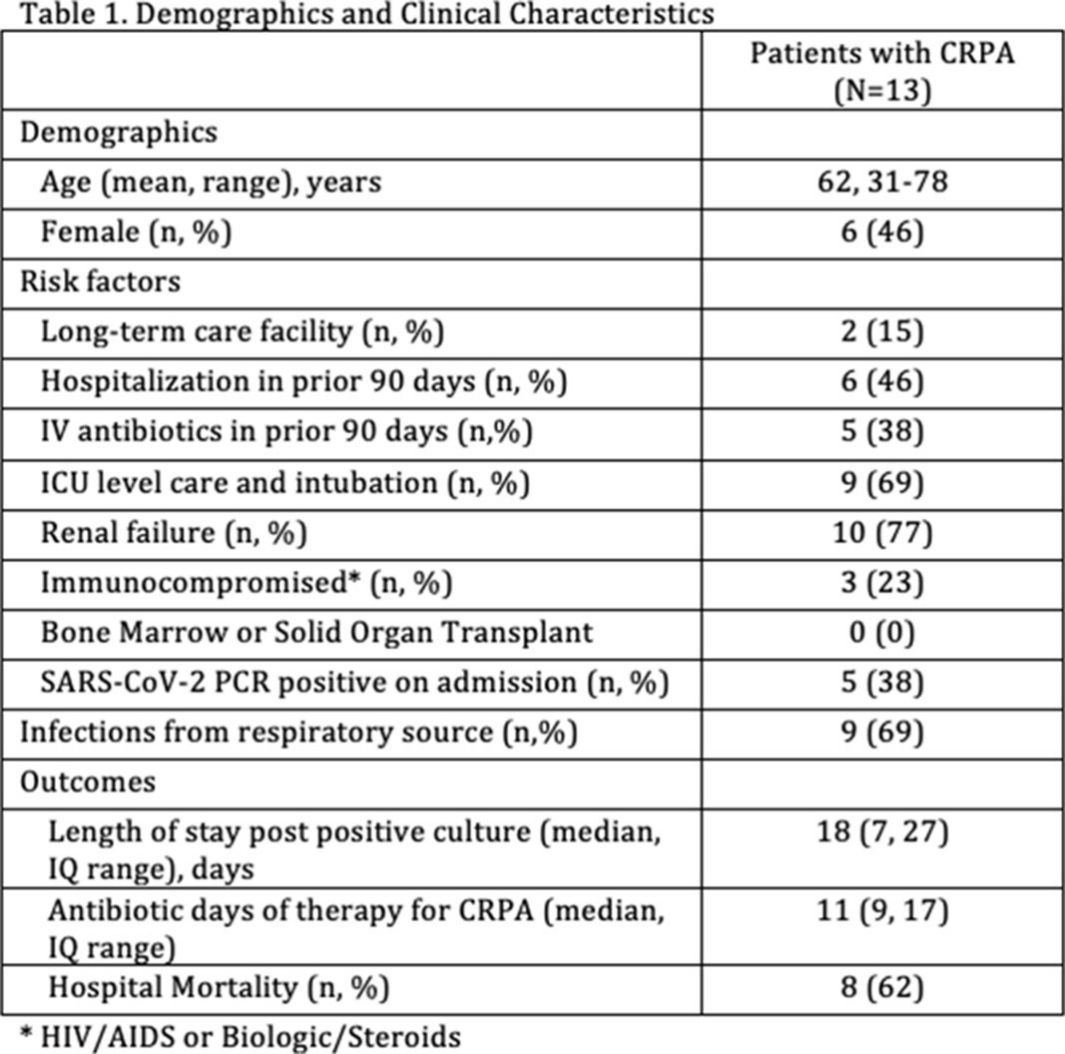# Stewardship Nightmare: Ceftolozane/Tazobactam–Resistant *Pseudomonas aeruginosa* Infections Accelerated by the COVID-19 Pandemic

**DOI:** 10.1017/ash.2021.137

**Published:** 2021-07-29

**Authors:** Adam Haviland, Gregory Weston, Priya Nori, Wendy Szymczak, Yi Guo, Rebecca M. Marrero Rolon

## Abstract

**Background:** The Centers for Disease Control and Prevention reported 32,600 cases, 2,700 deaths, and healthcare costs of 767 million dollars attributed to multidrug-resistant *Pseudomonas aeruginosa*. A recent study of 128 patients with nosocomial pneumonia due to *P. aeruginosa* showed the noninferiority of ceftolozane-tazobactam compared to meropenem. However, the resistance of ceftolozane-tazobactam due to AmpC mutations has been described. Compared with 2019, we observed an increase from 2 to 13 cases of ceftolozane-tazobactam–resistant *P. aeruginosa* (CRPA) during the COVID-19 pandemic at our institution in the Bronx, New York. **Methods:** A report of patients with CRPA between March and August 2020 was obtained. Data collected included demographics, hospitalization/IV antibiotic use in prior 90 days, SARS-CoV-2 PCR result, ICU admission, length of stay, antibiotic days of therapy, mortality, etc. **Results:** In total, 13 patients with CRPA infection were reviewed (Table 1). Among them, 2 patients were on the same inpatient medical-surgical unit but separated by 5 months. Also, 11 patients were from different medical-surgical units or ICUs. In addition, 5 patients (38%) were SARS-CoV-2 PCR positive. None of these COVID-19 patients were cohorted on the same unit, making horizontal spread of CRPA or COVID-19 unlikely. Finally, 8 of these patients died while hospitalized (4 were COVID-19 patients). **Conclusions:** We found a high incidence of mortality in patients with CRPA infection. Many patients had prolonged hospital stay and required ICU admission. Few patients were from long-term care facilities. Given the associated morbidity and mortality, increased surveillance and intensified antimicrobial stewardship efforts are needed to mitigate the impact of CRPA during the COVID-19 pandemic.

**Funding:** No

**Disclosures:** None

**Table 1.**